# From Invasive Species to Sustainable Nutrition: Safety, Nutritional, and Consumer Perception Study on *Faxonius limosus* in Serbia

**DOI:** 10.3390/foods14142523

**Published:** 2025-07-18

**Authors:** Milica Vidosavljević, Branislav Šojić, Tatjana Peulić, Predrag Ikonić, Jasmina Lazarević, Slađana Rakita, Milica Vidak Vasić, Zorica Tomičić, Ivana Čabarkapa

**Affiliations:** 1 Institute of Food Technology, University of Novi Sad, 21000 Novi Sad, Serbia; milica.vidosavljevic@fins.uns.ac.rs (M.V.); tatjana.peulic@fins.uns.ac.rs (T.P.); predrag.ikonic@fins.uns.ac.rs (P.I.); jasmina.lazarevic@fins.uns.ac.rs (J.L.); sladjana.rakita@fins.uns.ac.rs (S.R.); zorica.tomicic@fins.uns.ac.rs (Z.T.); 2 Faculty of Technology Novi Sad, University of Novi Sad, Bulevar Cara Lazara 1, 21000 Novi Sad, Serbia; sojic@tf.uns.ac.rs; 3 Laboratory for Building Ceramics, Institute for Testing of Materials IMS, Bulevar Vojvode Mišića 43, 11000 Belgrade, Serbia; milica.vasic@institutims.rs

**Keywords:** spiny-cheek invasive crayfish, *Faxonius limosus*, meat safety, nutritional quality, consumer survey

## Abstract

*Faxonius limosus* is an invasive alien crayfish species that has a negative effect on aquatic biodiversity. Using its meat as food could help reduce its ecological impact while providing a protein source. In order to do that, the initial step was to determine safety and nutritional parameters of crayfish meat. Samples from two localities were analyzed for energy value, moisture, ash, protein, fat, carbohydrates, fatty acid and amino acid composition, and macro- and micro-mineral content. Moreover, an online survey was conducted in order to evaluate the public’s current knowledge about invasive alien species and willingness to consume crayfish meat as a food product. Heavy metal concentrations (Hg, Pb, Cd) were below European Commission limits, confirming safety. The meat had a high protein content (16.68%), low fat (0.22%), and a favorable fatty acid profile with notable levels of omega-3 polyunsaturated fatty acids, particularly eicosapentaenoic acid (EPA) and docosahexaenoic acid (DHA). Predominant macro-minerals were K, Na, Ca, Mg, and P, while Zn, Cu, Fe, and Mn were the most abundant micro-minerals. Even though most participants (79.7%) were not informed about *Faxonius limosus*, the majority expressed willingness to participate in the assessment of new products made from invasive crayfish. These findings suggest that *F. limosus* meat is a nutritionally valuable and safe alternative protein source, with potential for sustainable food production and ecological management.

## 1. Introduction

Invasive alien species (IAS) have been identified as one of the most significant threats to humanity in the coming decade. According to the Global Assessment Report on Biodiversity and Ecosystem Services [1], they are also among the top five direct drivers of biodiversity loss. One of them, *Faxonius limosus*, formerly known as *Orconectes limosus*, is one of the most important aquatic invaders in European inland waters, recorded in as many as 22 European countries [2,3]. *Faxonius limosus* is present in upstream, middle, and downstream sections of the Danube River. In the upstream section, its presence has been recorded in Germany, Austria, and Slovakia. In the middle section, the species occurs in Hungary, Slovakia, Croatia, and Serbia. In the downstream section, it has been reported in Serbia, Bulgaria, Romania, and Ukraine [4,5,6,7,8,9,10,11]. In Serbia, it was first identified in 2002 in the Danube River near Apatin [3]. Since then, it has rapidly spread both upstream and downstream in the Danube River and its major tributaries but also throughout Sava, Tisa, Velika Morava, and Tamiš [4,12]. Commonly known as the spiny-cheek crayfish, *Faxonius limosus* is included on a list of IAS of universal concern within EU regulations. Moreover, it is one of the six North American crayfish species listed as “Old Non-Indigenous Crayfish Species” in Europe, recognized for its highly invasive and detrimental impact [13]. Because of its early maturation, high fecundity, high tolerance to a wide range of environmental conditions, omnivorous feeding, competitive behavior, better condition indices, and lack of native predators, *Faxonius limosus* has high invasive potential [2,13]. Its negative impact on native crayfish species is reflected by its competitive behavior and ability to carry crayfish plague, which is lethal to native crayfish [8]. Furthermore, its burrowing activity can destabilize riverbanks and cause economic damage [13]. Based on the facts mentioned above, it is evident that the spiny-cheek crayfish represents a considerable threat to aquatic biodiversity.

From a nutritional standpoint, several studies [14,15,16,17,18] have consistently reported that crayfish meat is highly nutritious, characterized by a high protein content. Śmietana et al. [15] reported that crayfish meat has a better nutritional protein quality in comparison to the FAO/WHO/UNU standards [15]. Additionally, crayfish meat contains low fat levels and exhibits a favorable fatty acid profile. For instance, Stanek et al. [17] demonstrated that the fatty acid composition of crayfish lipids is dominated by polyunsaturated fatty acids (PUFAs), which are known for their beneficial effects on human health. These nutritional attributes support the hypothesis that crayfish could serve as a sustainable and health-promoting food source. Also, aquatic animal foods represent a rich source of essential minerals and trace elements, even better than most terrestrial meat [19]. In the recent work of Śmietana et al. [15], it was indicated that crayfish meat could be an alternative to livestock meat, as it is a very good source of Ca, K, Mg, Na, P, and Cu, which are very important because these nutrients cannot be synthesized by the human body but must be consumed through food [20,21].

Considering the invasiveness of *Faxonius limosus* and the great threat it poses to native crayfish populations and biodiversity, prevention and control of this and similar crayfish represent the greatest challenge. On the other hand, recent data indicate that due to the increasing global population, the demand for protein is expected to double by 2050, and thus, identifying and exploring new sustainable protein sources and protein-rich foods has become imperative [22,23,24]. Given the high protein content of crayfish meat and its wide distribution within Serbia, Europe, and the world, the creation of innovative, eco-friendly and sustainable food products and animal feed from *Faxonius limosus* can be one of the solutions to the challenges in protein supply faced by the global population. Prevention of biodiversity loss caused by *Faxonius limosus* can be achieved by transforming this invasive species into a variety of eco-product and creating novel sustainable food. Therefore, it is essential to examine crayfish meat as a potential raw material by determining its safety and nutritional profile.

In order to determine the safety of crayfish meat, one of the most important things is to analyze heavy metals. The Danube River Basin collects water from 19 countries and is home to 115 million inhabitants, which means it can be polluted by various chemicals, including heavy metals. Moreover, industrialization and urbanization, as well as climate change, contribute to the bioaccumulation of heavy metals. As a result of higher temperature in aquatic systems, chemical reactions are accelerated, which result in increased solubility and bioavailability of heavy metals. Since they are not biodegradable, they accumulate over time. Join Danube Survey’s latest report confirms higher mercury levels than the Environmental Quality Standard [25,26].

Thus, this study aimed to determine the safety parameters and nutritional quality of *Faxonius limosus* meat as a potential raw material for new, innovative, and sustainable food products for both humans and animals. In addition, no data are currently available on consumer acceptance of spiny-cheek crayfish as a novel meat and protein source or on the consumption of products made from this meat. Therefore, an additional aim of the study was to assess consumer perception and willingness to consume crayfish-based products.

## 2. Materials and Methods

### 2.1. Sampling

Spiny-cheek crayfish individuals were caught at two locations along the Danube River: the first at Begeč (Novi Sad) and the second at Stari Slankamen (Inđija). Specimens of *F. limosus* were sampled monthly from April to September. The sampling procedure involved placing traps at suitable locations in the river for 24 h, at depths ranging from 1 to 8 m, in areas with slower currents. At each selected site, a series of five traps was set. Specimens of native crayfish species captured during sampling were identified on site and immediately returned, unharmed, to their natural habitat. The design of the crayfish traps is described in the work of Lazarević et al. [14].

After sampling, spiny-cheek crayfish were transported in containers filled with water taken from the sampling point to the Institute of Food Technology in Novi Sad. To maintain the health and survival of individuals, the containers were equipped with an aeration pump and a cooling system. Subsequently, individuals were humanely and non-invasively euthanized using a low-temperature (freezing) method in a Primax B051thermal shock chamber at −40 °C (Primax S.r.l., San Vito al Tagliamento, Italy) [27]. The samples were deep-frozen and stored at −18 °C until further analysis. Before the analysis, the crayfish were cleaned and deshelled, and the meat was homogenized.

### 2.2. Determination of Safety and Nutritional Quality

#### 2.2.1. Heavy Metals

After dry ashing, atomic absorption spectrometry was used to analyze heavy metals (Pb, Hg, Cd, and As), according to the EN method [28]. For Hg analysis in crayfish meat samples, the Advanced Mercury Analyzer AMA 254 (Altec, Prague, Czech Republic) was used. The results are expressed as mg of mineral content per 100 g of meat samples.

#### 2.2.2. Biogenic Amines

Six biogenic amines were extracted according to the following procedure. An appropriate amount of internal standard was added to nearly 2.00 g of each sample and homogenized with 10 mL 0.4 M perchloric acid using an Ultraturrax blender, after which the homogenate was centrifuged at 3000 rpm for 10 min, and supernatant was filtered into a 25 mL bottle. Extraction was repeated with 10 mL 0.4 M perchloric acid solution, mixed with a vortex, and centrifuged as before. Supernatants were combined and adjusted to 25 mL with 0.4 M perchloric acid. A 1.0 mL combined extract was made alkaline by adding 200 µL 2 M NaOH and buffered with 300 µL saturated NaHCO_3_. To this mixture, 2.0 mL of dansyl chloride solution was added, and the reaction mixture was incubated at 40 °C for 45 min. Residual dansyl chloride was removed by adding 100 µL of ammonia. After 30 min, the mixture was adjusted to 5.0 mL with acetonitrile, filtered (0.45 mm, PTFE, MS Springer filter), and analyzed [29,30].

#### 2.2.3. Macro- and Micro-Minerals

The contents of macroelements (K, Ca, Mg, Na, P) and microelements (Fe, Mn, Cu) were analyzed using the ISO method, which is based on atomic absorption spectrometry [31]. The analysis was performed with a flame atomic absorption spectrometer (Varian SPECTRA AA-10, Varian Techtron Pty Limited, Mulgrave, Victoria, Australia) equipped with a flame furnace, utilizing an air-acetylene flame. Before the measurement, the samples were mineralized by dry ashing.

#### 2.2.4. Chemical Composition

The crude ash content and moisture were determined according to ISO methods [32,33]. After acid hydrolysis, the fat content was determined using Soxhlet extraction [34]. The total phosphorus content expressed as P_2_O_5_ was performed according to ISO methods [35]. The Kjeldahl method was used to assess the protein content using a nitrogen-to-protein conversion factor of 6.25 [36]. The carbohydrate content was calculated based on the formula provided by Omole et al. [37] as 100% minus the sum of crude protein, crude fat, crude ash, and moisture percentages. The sugar content was determined using the Luff–Schoorl method [38]. The gross energy value (Kcal/100 g sample) was estimated by multiplying the crude fat (9 Kcal/g), protein (4 Kcal/g), and carbohydrate (4 Kcal/g) contents, as outlined in the EU regulations [39].

#### 2.2.5. Amino Acid Composition

Ion exchange chromatography was used for the determination of the amino acid profile of crayfish abdominal meat using an automatic Amino Acid Analyzer Biochrom 30+ (Biochrom, Cambridge, UK), [40]. Samples were previously hydrolyzed in 6M hydrochloric acid (Merck, Germany) at 110 °C for 24 h. After hydrolysis, the samples were cooled to room temperature and dissolved in 25 mL of loading buffer at pH 2.2 (Biochrom, Cambridge, UK). Subsequently, prepared samples were filtered through a 0.22 µm PTFE filter (Plano, TX, USA) into a vial (Agilent Technologies, Santa Clara, CA, USA) and stored in a refrigerator prior to analysis. The technique was based on amino acid separation using strong cation exchange chromatography, followed by the ninhydrin color reaction and photometric detection at 570 nm, except for proline, which was detected at 440 nm. By comparing the retention time of amino acids with the retention times of standard amino acids purchased from Sigma Aldrich (Amino Acid Standard Solution (Sigma-Aldrich, St. Louis, MI, USA)), the amino acid peaks were identified. The results were presented as grams of amino acid per 100 g of sample. Tryptophan was not determined in the analysis due to its degradation during acid hydrolysis, which limits the accuracy of its quantification.

#### 2.2.6. Fatty Acid Composition

Total lipids from crayfish meat were extracted using a chloroform/methanol mixture (2:1, *v*/*v*) under continuous agitation for 2.5 h at room temperature [41]. The extract was filtered through filter paper and evaporated to dryness. Fatty acid methyl esters (FAME) were then prepared via transmethylation with a 14% boron trifluoride–methanol solution, following the ISO 12966-2 method [42]. FAME analysis was performed using an Agilent 7890A gas chromatograph (Agilent Technologies, Santa Clara, CA, USA) equipped with a Supelco SP-2560 fused silica capillary column (100 m × 0.25 mm, d = 0.20 µm) (Supelco, Bellefonte, PA, USA) and a flame ionization detector (FID). Helium served as the carrier gas, with injector and detector temperatures set at 250 °C. The column temperature program followed the method described by Lazarević et al. [14]. FAME identification was based on the comparison of retention times with those of the Supelco 37 FAME Mixture reference standard. The results were expressed as the relative percentage of each identified FAME in total FAME.

#### 2.2.7. Statistical Analysis

All data were presented as the mean value ± standard deviation (SD). Statistical analysis of the data was performed using the software package Statistica, version 14 (TIBCO Software Inc., Santa Clara, CA, USA, 2020) (Addinsoft, New York, NY, USA). Statistical analysis of the data, including significant differences and Pearson’ correlations at a 0.05 significance level for all variables, was performed using a one-way ANOVA, followed by Tukey’s HSD multiple comparison tests. For post-hoc comparisons, Tukey’s HSD test was employed to determine statistically significant differences between groups. This test was chosen due to its strong control over the family-wise error rate, which was considered important given the number of pairwise comparisons conducted. Although conservative, it ensures robustness and reproducibility of the results, which aligns with the objectives of this study [43].

All analyses were performed in five replicates, except for amino acid and fatty acid determinations, which were conducted in triplicate.

### 2.3. Consumer Survey

The consumer survey was conducted in Serbia as an online questionnaire on a sample of 315 respondents. The socio-demographic characteristics, which include gender, age, education, place of residence, employment, and family structure of individuals, are represented in Table 7. The questionnaire was developed based on a comprehensive literature review, adapted to the subject of the examination, and consisted of sixteen questions divided into three sections [44,45,46,47]. The first part included three questions regarding respondents’ exposure to science. The second part consisted of seven questions with the main goal of assessing awareness and understanding of biodiversity conservation, particularly regarding invasive species, like the spiny-cheek crayfish in the Danube River. The third section included six questions focused on evaluating consumer interest and behavior related to river products, particularly those derived from the spiny-cheek crayfish. Three questions used a 5-point Likert scale with response options labelled as follows: Yes, I would be happy to participate/Yes, very gladly; Probably yes; Not sure/Neutral; Probably no; No, I would not.

To examine the influence of socio-demographic factors on consumers’ responses to the questions, Spearman’s rank correlation coefficient was used. Additionally, correlations within groups of questions were analyzed. Spearman’s rank correlation measures the strength and direction of linear relationships between variables, with values ranging from −1 (perfect negative correlation) to +1 (perfect positive correlation). Correlations were considered statistically significant at a *p*-value of <0.05. Only statistically significant correlations were discussed.

The correlation results were visualized using a heatmap, which graphically represents the magnitude and direction of correlations between variables, enabling a clearer interpretation of the observed patterns. All analyses were performed using the software package Statistica, version 14 (TIBCO Software Inc., Santa Clara, CA, USA, 2020) (Addinsoft, New York, NY, USA) and Excel programs.

### 2.4. Ethical Statement

The ethical statement was obtained from the Ethics Committee for Protection and Welfare of Experimental Animals of the University of Novi Sad, Serbia (EK: II-2022-03) and from the Ethics Committee of the Institute of Food Technology in Novi Sad No. 175/I/22-3. The committee’s opinion is based on the Rulebook (Annex 4 of the “Official Gazette of RS”, No. 39/10), which explicitly states that ethical approval is not required for research involving invertebrate animals.

## 3. Results and Discussion

### 3.1. Determination of Safety and Nutritional Quality

#### 3.1.1. Heavy Metals

One of the main focuses of this work was to evaluate the safety of crayfish meat, as a potential food source. Due to their constant contact with bottom sediments, *Faxonius limosus* poses a high risk of bioabsorption and bioaccumulation of heavy metals. Therefore, they are considered as a bio indicator of environmental pollution. As humans are primarily exposed to heavy metals through food and since crayfish are considered a potential food source, the first necessary step of this work was to determine the heavy metal content in crayfish meat [14]. The contents of 4 heavy metals, cadmium (Cd), mercury (Hg), lead (Pb), and arsenic (As), which are classified as toxic elements [48], in spiny-check crayfish meat from two locations, Stari Slankamen and Begeč, are presented in Table 1. The contents of Hg, Pb, and Cd were below the maximum limits established by the European Commission [49,50,51]. Also, it was below the limit set by the legislation of the Republic of Serbia [52]. As for As, its maximum content is currently not defined by the aforementioned rulebooks, but according to the European Food Safety Authority (EFSA), food is one of the main sources of As exposure, which is why we measured its concentration. [53]. As can be seen, its concentration from both examined locations was below 0.1 mg/kg. Similar results were found by Jayakody et al. [54] for the total As content in freshwater giant river prawn (0.036–0.051 mg/kg). It is worth noting that, Śmietana et al. [15] determined that the concentration of Cd and Pb in the abdomen of crayfish meat from Lake Sominko (Poland) was higher than in the chela but did not exceed the maximum allowed limit. They explain that it is a result of different biological and natural factors. From the perspective of food safety, the concentration of heavy metals below the legally permitted limits is of significant importance, ensuring that crayfish meat can be used as a raw material in food product manufacturing.

#### 3.1.2. Biogenic Amines

The concentrations of six biogenic amines in raw crayfish meat are presented in Table 2. As can be seen from the results, biogenic amines were not detected in any of the analyzed samples, either from Stari Slankamen or from Begeč. It should be specifically noted that histamine was not detected, as it is considered the most important biogenic amine from a toxicology point of view. Also, it is the only biogenic amine with legally defined limits in Europe—100 mg/kg in some fish species and 200 mg/kg in fishery products, according to EC Regulation 2073/2005 [55].

Determination of biogenic amines in food, particularly in meat and meat products, is essential, as they serve as indicators of quality and good hygiene practices, with putrescine and cadaverine specifically recognized in this role [30].

#### 3.1.3. Macro- and Micro-Minerals

One of the six nutrients required to maintain human health are minerals. They are very important for human growth, development, and metabolism [20,21]. Even though Fe, Zn, Cu, and Mn have many biological roles in biochemical processes, enzymatic activities, and are essential for the proper functioning of the human body, they are toxic in higher quantities [56,57], and their determination is necessary. The content of macro- and micro-minerals in crayfish meat is presented in Table 3. As observed, the predominant macro-mineral in the meat from Stari Slankamen and Begeč was K (1524.50 mg/kg; 1625 mg/kg), followed by Na (1047.57 mg/kg; 1067 mg/kg), Ca (603.15 mg/kg; 579.56 mg/kg), Mg (229.06 mg/kg; 229.72 mg/kg), and P (2957.5 mg/kg; 3560 mg/kg). There was no significant difference (*p* > 0.05) in the macro-mineral content between the two examined locations. Lazarević et al. [14] reported similar contents of macro-minerals, except for P, in which the content was higher than that found in this study. Furthermore, the predominant micro-mineral in the meat from Stari Slankamen and Begeč was Zn (22.36 mg/kg; 16.60 mg/kg), followed by Cu (14.07 mg/kg; 10.21 mg/kg), Fe (10.70 mg/kg; 9.69 mg/kg), and Mn (1.76 mg/kg; 1.49 mg/kg). There were no significant differences (*p* > 0.05) in the contents of micro-minerals between the two examined locations.

#### 3.1.4. Chemical Composition

In order to explore the potential of crayfish as raw material for new, innovative, and sustainable products for both humans and animals, the chemical composition of the meat from two locations, was analyzed, and the results are presented in Table 4.

Regarding the basic chemical composition, no significant differences (*p* > 0.05) were found between different tested locations, which was in line with expectations. As shown in Table 4, crayfish meat is high in protein (average values from both locations of 16.68%) comparable to shrimp, crab, marine and freshwater fish, pork, beef, and poultry meat [58,59,60,61,62]. In contrast, it has a low fat content of 0.20% and 0.23% in samples from Stari Slankamen and Begeč, respectively. Due to its low fat and high protein content, this meat can be considered lean [30], making it suitable for hypocaloric human diets and as an alternative protein source in animal food and feed [14,15].

Furthermore, it has a high moisture content of approximately 79.98% (average value from both locations). The average ash content from both locations was 1.27%, and the gross nutritional value was 322.9 kJ/100 g. Similar findings have been reported by other authors, who also characterized crayfish meat by its high protein content, low fat content, and favorable fatty acid profile [14,15,16,17,20,37,61,63].

In addition to its high nutritional value, crayfish meat is highly appreciated in several cultures due to its excellent culinary properties, with many consumers regarding it as a delicacy comparable to caviar [20,64,65].

#### 3.1.5. Amino Acid Profile

The amino acid profile of the examined crayfish meat from two locations is presented in Table 5. The levels of proline and alanine differed significantly (*p* ≤ 0.05) between the locations. Lysine was the predominant essential amino acid (1.61 g/100 g in crayfish meat from Stari Slankamen and 1.64 g/100 g in crayfish meat from Begeč), while glutamic acid was the predominant non-essential amino acid in samples from both locations (2.51 g/100 g and 2.77 g/100 g in crayfish meat from Stari Slankamen and Begeč, respectively). The three most abundant amino acids in crayfish meat from Stari Slankamen and Begeč were glutamic acid (2.51 g/100 g and 2.77 g/100 g), aspartic acid (1.94 g/100 g and 2.07 g/100 g), and lysine (1.61 g/100 g and 1.64 g/100 g).

Differences in amino acid profiles from two locations can be explained as the result of different growing environments. Salinity, water temperature, organic matter, dissolved oxygen, soil, and sediment are just some of the factors that can affect protein metabolism in crayfish, leading to fluctuation in amino acid profiles from different locations [20].

**Figure 1:**
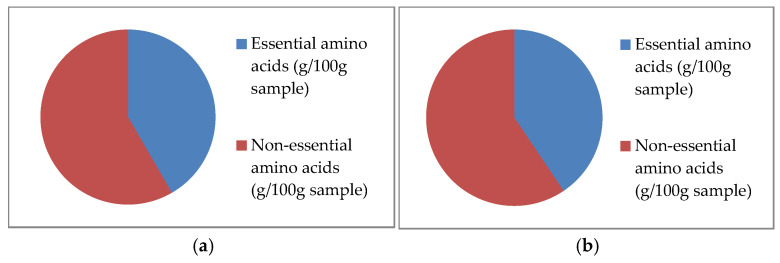
Essential and non-essential amino acids in spiny-cheek crayfish from Stari Slankamen (**a**) and Begeč (**b**).

The proportion of essential and non-essential amino acids in the examined samples is shown in Figure 1. As can be seen, crayfish meat from Stari Slankamen has a slightly higher content of essential amino acids (6.88 g/100 g sample) compared to Begeč (6.78 g/100 g sample). Moreover, no significant difference in the total amino acid content was determined (*p* > 0.05), with crayfish meat from Stari Slankamen containing 16.50 g/100 g and Begeč containing 16.73 g/100 g.

It should be emphasized that lysine was the most abundant essential amino acid in the meat of *Faxonius limosus*. This finding is particularly relevant, as lysine is often the first limiting amino acid in cereal-based diets in humans and in monogastric animals [66,67]. Therefore, the inclusion of crayfish meat as an alternative protein source may contribute to a more balanced amino acid intake.

#### 3.1.6. Fatty Acid Profile

A total of 20 fatty acids were detected in crayfish meat from Stari Slankamen and 14 from Begeč. The fatty acid profile of crayfish meat for both locations is presented in Table 6. There were no significant differences (*p* > 0.05) in the content of the following fatty acids in the samples from Stari Slankamen and Begeč: palmitic acid (16.5 and 18.30 g/100 g), palmitoleic acid (4.64 and 5.25 g/100 g), heptadecanoic acid (1.07 and 1.03 g/100 g), alpha-linolenic acid (1.27 and 2.13 g/100 g), eicosadienoic acid (1.97 and 1.36 g/100 g), and docosahexaenoic acid (3.62 and 3.9 g/100 g). Moreover, total monounsaturated fatty acids (MUFA) were similar between crayfish meat from Stari Slankamen and Begeč (27.28 and 23.02 g/100 g total fatty acids respectively).

**Figure 2:**
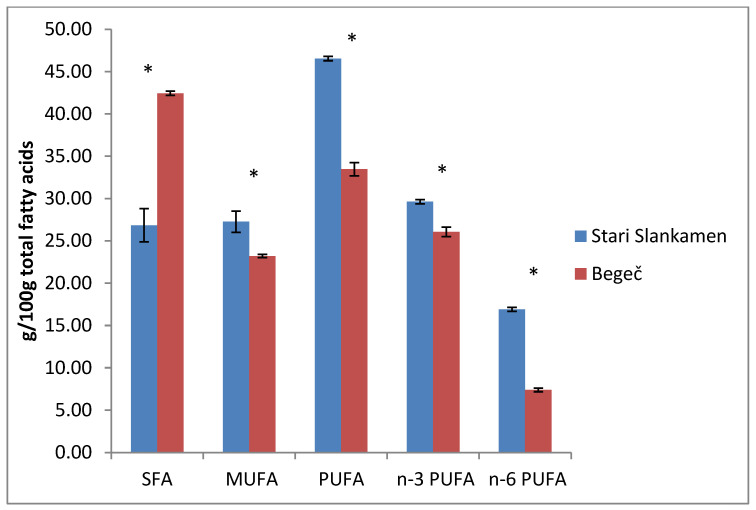
The contents of SFA, MUFA, PUFA, n-3 PUFA, and n-6 PUFA from spiny-cheek crayfish meat. Bars represent mean values ± standard error. An asterisk (*) indicates a statistically significant difference between samples (*p* < 0.05). Abbreviations: SFA, Total saturated fatty acids, MUFA, Total monounsaturated fatty acids, PUFA, Total polyunsaturated fatty acids, n-3 PUFA, Total omega-3 fatty acids, n-6 PUFA Total omega-6 fatty acids. ND, Not detected.

Crayfish meat from Begeč had a higher saturated fatty acid (SFA) content (42.45 and 26.84 g/100 g) but lower polyunsaturated fatty acid (PUFA) content (33.47 and 46.54 g/100 g). Furthermore, both n-3 PUFA (26.08 and 29.63 g/100 g) and n-6 PUFA (7.39 and 16.91 g/100 g) levels were lower in crayfish meat from Begeč. The content of SFA, MUFA, PUFA, n-3 PUFA, and n-6 PUFA for both locations is shown in Figure 2. Furthermore, the (n-6)/(n-3) fatty acid ratios of 0.57 and 0.28 for samples from Stari Slankamen and Begeč indicate an exceptionally favorable fatty acid profile. These values are well below the recommended upper limit of 4:1, suggesting a high proportion of omega-3 fatty acids, which are known for their anti-inflammatory and cardioprotective properties [68]. Omega-3 polyunsaturated fatty acids (n-3 PUFAs), especially eicosapentaenoic acid (EPA) and docosahexaenoic acid (DHA), play a vital role in human health by influencing numerous physiological functions. These long-chain fatty acids contribute to normal brain function and exhibit well-documented cardioprotective and anti-inflammatory properties [69]. Therefore, it is worth mentioning that the contents of EPA and DHA in samples from Stari Slankamen were 24.72 g/100 g and 3.62 g/100 g, respectively, while in samples from Begeč, they were 20.05 g/100 g and 3.9 g/100 g, respectively.

In the work by Li et al. [70], twenty-nine species—including wild and cultured freshwater and marine fish and shrimp—were examined. The study concluded that marine fish and shrimp had much higher total n-3 PUFA levels than n-6 PUFA, while most freshwater fish and shrimp showed much lower total n-3 PUFA levels compared to n-6 PUFA. The omega-3 fatty acid content in crayfish meat was comparable to levels found in marine fish, particularly in white herring (26.4%), largehead hairtail (28.4%), Bluefin leatherjacket (29%), Spanish mackerel (26.6%), and Chinese silver pomfret (25.3%).

Despite the very low fat content in the analyzed samples (0.20% and 0.23%), the favorable fatty acid profile is of great importance for both human and animal health. Based on these findings, it can be concluded that the fatty acid profile of meat from Stari Slankamen was more favorable due to its higher polyunsaturated fatty acid content. Differences in n-3 and n-6 PUFA may result from variations in diet, as these fatty acids can only be synthesized de novo by plants [63].

Stanek et al. [63] compared the fat content and fatty acid profile of the spiny-cheek crayfish meat caught from the Brda River and Lake Gopło. They also concluded that while fat content remained similar, the fatty acid profile showed significant differences. Furthermore, in another study from Stanek et al. [65], the authors reported that the total fat content was independent of sex, whereas the contents of SFA, MUFA, and PUFA differed significantly between males and females. Based on everything that was mentioned, it can be concluded that the difference in fatty acid profile is a result of different diets and sex.

### 3.2. Consumer Survey

#### 3.2.1. Socio-Demographic Characteristics—Spearman’s Rank Correlation Coefficients Between Q1 and Q7

The data in Table 7 indicate that the majority of participants (74%) were female. Most of the respondents were aged between 25 and 34 years (45.71%) and had a postsecondary education (79.7%). The smallest share was respondents above 65 years (0.63%). Regarding the place of residence, 88.3% lived in a city with the Danube River flowing through it (79.7%). The respondents were mainly employed (83.5%). As for family structure, 30.2% of them were below 27 years old, living with a partner and child/children, and 25.1% answered that they live alone.

A limitation of this study is the significant difference in the ratio of male to female consumers among the participants. This gender imbalance could potentially introduce bias and affect the generalizability of the results. Future studies should aim for a more balanced sample to better represent the population and minimize gender-related bias.

**Table 7:** Socio-demographic characteristics.

Question Number	Socio-Demographic Characteristics	n	Share (%)
Q1	Gender		
	Male	82	26
	Female	233	74
Q2	Age		
	18–24	40	12.70
	25–34	144	45.71
	35–44	69	21.90
	45–64	60	19.05
	>65	2	0.63
Q3	Education		
	Elementary	0	0
	Secondary	64	20.3
	Postsecondary	251	79.7
Q4	Employment		
	Volunteer	2	0.6
	Student	32	10.2
	Unemployed	11	3.5
	Employed	263	83.5
	Pensioner	3	1
	Other	5	1.6
Q5	Family structure		
	I live alone	79	25.1
	I am married and live with my partner without children	28	8.9
	I am under 27 years old and live with my partner and child/children	95	30.2
	I am 27 years or older and live with my partner and child/children	1	0.3
	Multigenerational family	58	18.4
	Other	54	17.1
Q6	Place of residence		
	Village	16	5.1
	Town	21	6.7
	City	278	88.3
Q7	Does the Danube River flow through your place of residence?		
	Yes	251	79.7
	No	64	20.3

Spearman’s rank correlation coefficients between socio-demographic characteristics are presented in Table 8. The strongest and most significant correlations are, in order: Q3–Q4, Q6–Q7, Q2–Q4, and Q3–Q7. According to these results, higher education does not necessarily imply employment; the respondents who live in a city, live in a city in which the Danube River flows; and older individuals are more often those who are employed. Q2 and Q3 are positively correlated, indicating that older respondents tend to have higher education levels. Among all the correlations, the weakest (i.e., statistically insignificant) is between gender and the size of the place of residence.

#### 3.2.2. Respondents’ Exposure to Science—Spearman’s Rank Correlation Coefficients Between Q12 and Q26

Table 9 presents information regarding the extent of respondents’ engagement with science. The majority of participants (68.6%) reported having a job that is, in some way, connected to scientific research or a scientific institution. Additionally, a significant share of respondents reported encountering science and scientific research via online platforms (50.2%) and scientific journals (40.6%). Furthermore, 35.9% reported engaging with science through reading scientific papers and books.

To gain a deeper understanding of respondents’ exposure to science, Spearman’s rank correlation coefficients were calculated for responses to questions Q12 through Q26 (Table 10). A 5-point Likert scale was used for questions Q13 to Q26 (1—very low, 5—extremely high). This set of questions revealed several noteworthy correlations, highlighting strong interrelations between different facets of scientific engagement.

Notably, a positive correlation was observed between following scientific literature and engaging in discussions on scientific topics (Q16–Q26). Similarly, keeping up with scientific journals was positively correlated with seeking information on the websites of relevant institutions (Q16–Q24; Q16–Q18). The data also indicate that scientists are more likely than other respondents to use the Internet as a primary source of scientific information, as reflected in a strong positive correlation between corresponding questions (Q16–Q17).

A negative correlation was observed between Q12 and Q24, as well as between Q12 and Q16, suggesting that individuals working in scientific institutions (Q12) tend to report lower levels of engagement with scientific literature (Q16 and Q24). This unexpected finding may imply that professional involvement in science does not necessarily translate to frequent reading of scientific literature or participation in related discussions, at least not in the way it was captured in this questionnaire. Interestingly, no correlation was found between watching television and reading scientific journals, which may suggest that individuals involved in scientific work tend to watch TV less frequently.

#### 3.2.3. Perception and Awareness of Biodiversity and Invasive Species in Serbia—Spearman’s Rank Correlation Coefficients Between Q31 and Q37

In Table 11, questions related to the perception and awareness of biodiversity and invasive species in Serbia are presented. The majority of respondents (79.4%) correctly identified the meaning of the term “invasive species”, yet most were not informed about the spiny-cheek crayfish (*Faxonius limosus*) (79.7%). Additionally, 39.3% stated that they do not know what effects this crayfish species may have, although 55.6% believe that the biodiversity of the Danube is under threat.

These findings suggest that while general awareness of the concept of invasive species is relatively high, there is a lack of specific knowledge regarding particular invasive species and their ecological impact. This indicates the need for more targeted educational efforts to improve public understanding of how invasive species affect biodiversity, especially in the context of the Danube ecosystem.

Spearman’s rank correlation coefficients between Q31 and Q37 are presented in Table 12. The positive correlation between Q34_1 and Q34_3, Q34_4, and Q34_5 indicates that respondents who believe that the invasive crayfish species contributes to the extinction of native crayfish (*Astacus astacus*, *Astacus leptodactylus*) are also likely to believe it causes the destruction of fish eggs and juveniles, transmits crayfish plague, and destabilizes riverbanks. Additionally, a positive correlation was found between Q34_3 and Q34_4, as well as between Q34_4 and Q34_5. This further supports the idea that respondents perceive multiple negative ecological effects associated with the presence of the spiny-cheek crayfish.

#### 3.2.4. Consumer Attitude Towards River and Crayfish Products—Spearman’s Rank Correlation Coefficients Between Q40 and Q45

Table 13 presents questions aimed at evaluating consumer interest and behavior related to river products, particularly those derived from the spiny-cheek crayfish. The findings indicate that most participants are either enthusiasts or occasional consumers of river products (79.4%), with a typical consumption frequency of once per week (71.4%). Among the river products consumed, trout was the most frequently mentioned (36.6%), followed by carp (31.7%), catfish (20.2%), no consumption of river products (9.8%), and river crayfish (1.6%). Regarding the tasting and purchase of crayfish meat or related products, most respondents conveyed a favorable outlook, suggesting a potential market demand for these offerings.

Spearman’s rank correlation coefficients between Q40 and Q45 are presented in Table 14. The strongest positive correlations are in the following order: Q43–Q44, Q44–Q45, Q43–Q45. These findings indicate that respondents willing to participate in a sensory assessment of newly developed river crayfish-based products are also likely to sample various crayfish dishes at a promotional gastronomic event and would be inclined to purchase such products if they were commercially available.

#### 3.2.5. Correlation Between Socio-Demographic Characteristics and Respondents’ Engagement in Science

**Figure 3:**
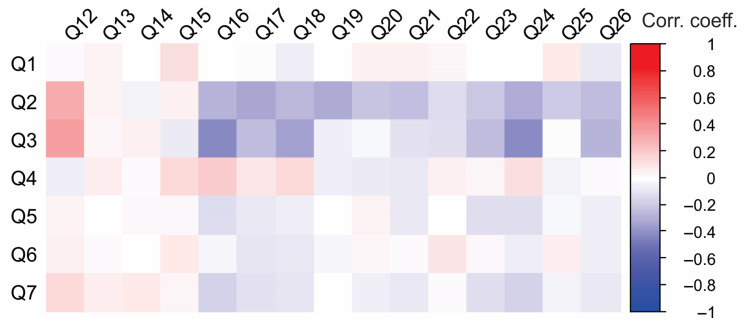
Correlations between socio-demographic characteristics and respondents’ engagement with science.

**Figure 4:**
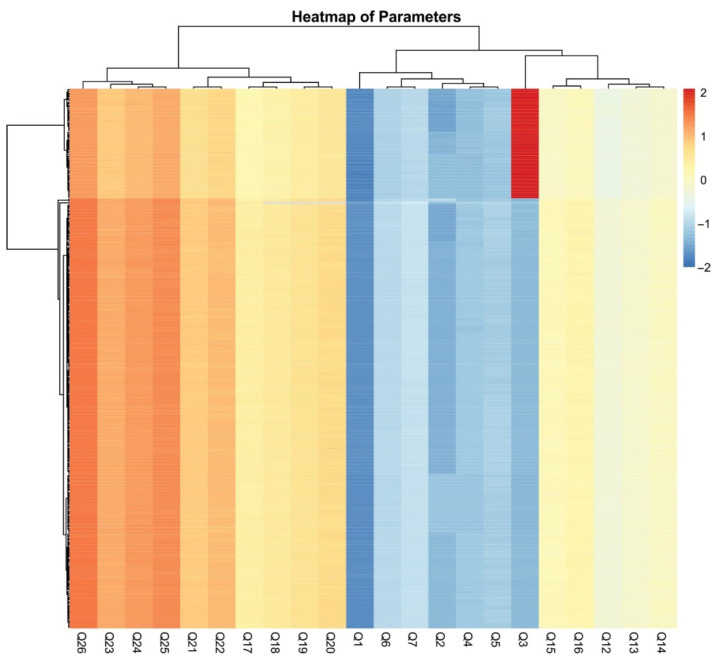
Heatmap of parameters showing correlations between socio-demographic characteristics and exposure to science.

Figure 3 presents the correlations between socio-demographic characteristics and respondents’ exposure to science. Gender and place of residence did not have a significant impact on respondents’ exposure to science. In contrast, age had the most notable effect on the responses. Older participants were more likely to have jobs related to scientific research or science institutions. Additionally, a higher level of education was associated with a greater likelihood of being employed in roles connected to scientific research or science institutions. To further emphasize these conclusions, a heatmap illustrating the correlations between socio-demographic characteristics and engagement with science provides a visual representation of the parameters (Figure 4). It is seen that professional background can be a determining factor—those working in technical or research-driven fields are likely to demonstrate greater interactions with scientific literature. Clusters of high correlation suggest targeted strategies for improving science communication and outreach among less engaged groups.

#### 3.2.6. Correlations Between Socio-Demographic Characteristics and Perception and Awareness of Biodiversity and Invasive Species in Serbia

**Figure 5:**
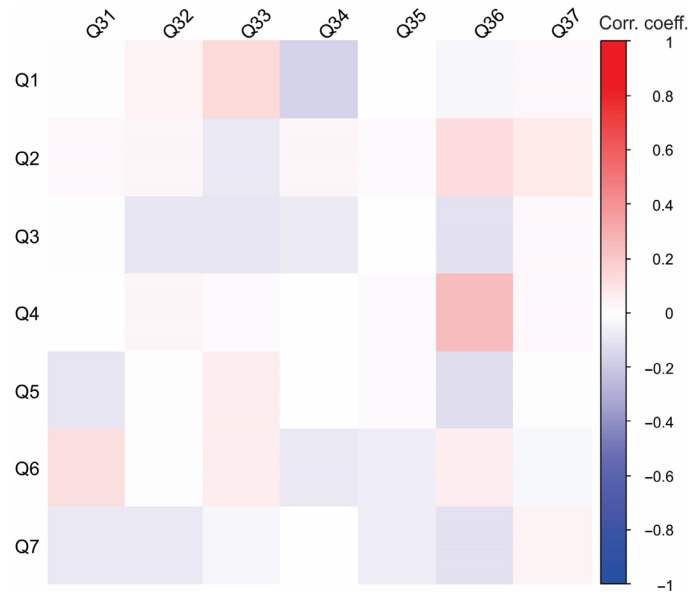
Correlations between socio-demographic characteristics and perception and awareness of biodiversity and invasive species in Serbia.

**Figure 6:**
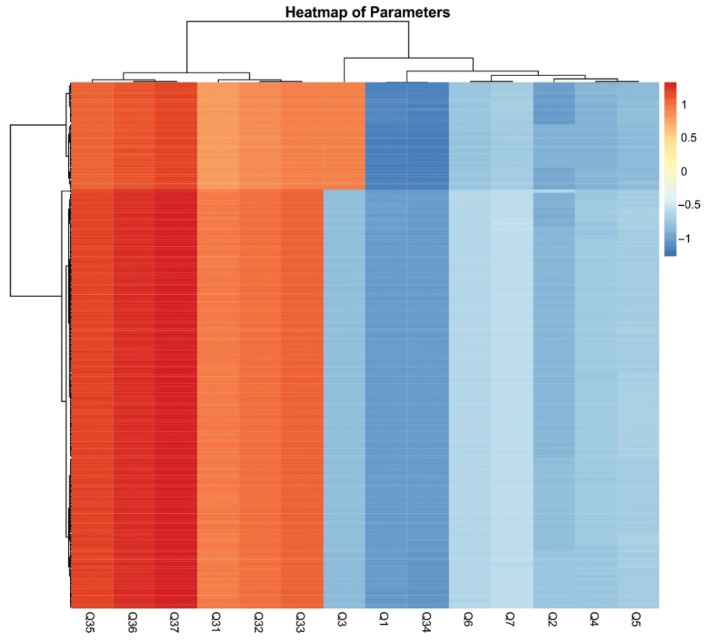
Heatmap of parameters showing correlations between socio-demographic characteristics and perception and awareness of biodiversity and invasive species in Serbia.

Figure 5 presents the correlations between socio-demographic characteristics and awareness of biodiversity and invasive species in Serbia. Only a few significant correlations were observed within this group of questions. The positive correlation between Q6 and Q31 suggests that urban respondents acknowledge ongoing discussions and initiatives to promote biodiversity conservation, yet they perceive these efforts as insufficient. Additionally, a positive correlation between Q1 and Q33 suggests that female respondents were generally less familiar with the spiny-cheek crayfish. A heatmap depicting correlations between socio-demographic characteristics and perceptions of biodiversity and invasive species in Serbia provides valuable insights into public awareness and attitudes toward environmental issues (Figure 6). For instance, younger respondents or those with higher education levels may exhibit stronger awareness of invasive species and ecological threats, likely due to exposure through academic studies or digital media. Conversely, individuals in rural areas may demonstrate a more direct familiarity with local biodiversity and invasive species based on firsthand interactions with ecosystems. The employment sector could also play a role, with those in scientific or environmental fields showing a deeper understanding of conservation challenges.

#### 3.2.7. Correlation Between Socio-Demographic Characteristics and Consumer Attitude Towards River and Crayfish Products

**Figure 7:**
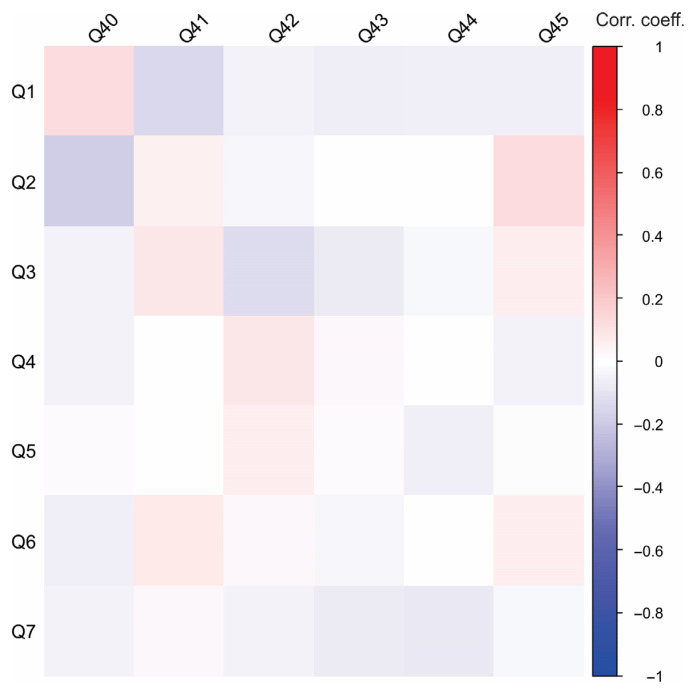
Correlations between socio-demographic characteristics and consumer attitude towards river and crayfish products.

**Figure 8:**
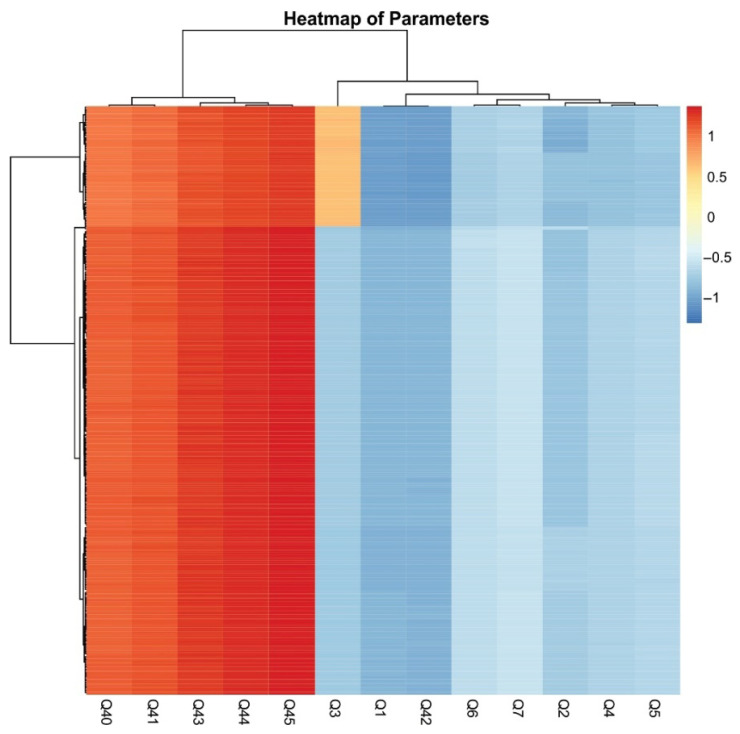
Heatmap of parameters showing correlations between socio-demographic characteristics and consumer attitudes towards river and crayfish products.

Figure 7 presents the Pearson’s correlations between socio-demographic characteristics and consumer attitudes toward river and crayfish products. Employment status, family structure, place of residence, and whether the Danube River flows through the participant’s place of residence did not significantly affect attitudes toward these products. The negative correlation between Q2 and Q40 suggests that older participants have a higher tendency to consume fish products. In contrast, a positive correlation between age and willingness to try a new product based on river crayfish meat suggests that younger individuals are more open to experimenting with novel products. These findings contribute to a better understanding of demographic trends in openness toward crayfish-based products. The heatmap depicting correlations between socio-demographic characteristics and consumer attitudes toward river and crayfish products provides valuable insights into purchasing behaviors, preferences, and market potential (Figure 8).

## 4. Conclusions

The results of this study demonstrate that crayfish meat obtained from two locations in Serbia is both safe for consumption and possesses high nutritional value. No significant differences were observed between the two sampling sites with respect to safety parameters and overall chemical composition. Concentrations of heavy metals (Hg, Pb, As, and Cd) in all analyzed samples were within the permissible limits defined by relevant regulatory standards. Additionally, none of the tested biogenic amines were detected.

Regarding mineral composition, K and Zn were identified as the predominant macro- and microelements, respectively. The crayfish meat was characterized by a high protein content and low fat content. Statistically significant differences were not noticed in the content of essential amino acids between samples from two locations. Differences were found in fatty acid profiles; samples from Stari Slankamen had a higher proportion of polyunsaturated fatty acids. It should be noted that crayfish meat has a high percentage of omega-3 polyunsaturated fatty acid, especially EPA, and favorable (n-6)/(n-3) fatty acid ratios. These findings suggest that crayfish meat represents a promising, novel, and sustainable raw material for both human and animal products.

In addition, a consumer survey was conducted to assess perceptions and willingness to consume crayfish-based products. The results revealed that although general awareness of invasive species is relatively high, there is limited specific knowledge about *Faxonius limosus*. This indicates the need for targeted educational initiatives to enhance public understanding of the ecological impacts of invasive species, particularly within the Danube River ecosystem. As for the willingness to consume crayfish-based products, younger respondents were more open to experimenting with novel products. These findings contribute to a better understanding of demographic trends in openness toward crayfish-based products.

Based on these findings, future research should prioritize the development of innovative and sustainable food and pet food products derived from *Faxonius limosus*. Leveraging its nutritional potential could facilitate the formulation of high-quality, protein-rich products tailored to meet the dietary requirements of both humans and animals.

This study demonstrated that a huge amount of shell remains following meat extraction. Consequently, future investigations should focus on efficient valorization and utilization strategies for this by-product. To advance the zero-waste approach, subsequent research will explore the potential of the shells for the recovery of high-value compounds, such as chitin and antioxidants, as well as the development of innovative biodegradable and edible packaging materials derived from shell biomass. These valorization pathways not only enhance resource efficiency but also align with the broader goals of the circular economy and environmental sustainability.

Moreover, targeted initiatives are essential to raise consumer awareness regarding the nutritional and environmental benefits of crayfish-based products. Public education efforts could significantly improve acceptance of these novel products, especially among older adults and pet owners who prioritize healthier and more eco-conscious choices.

## Figures and Tables

**Table 1:** Heavy metal content in spiny-cheek crayfish meat.

Heavy Metals (mg/kg)	Stari Slankamen	Begeč	Maximum Legally Permitted Values (mg/kg Wet Weight)
Hg	0.20 ± 0.07 ^a^	0.24 ± 0.03 ^a^	0.50
Pb	<0.1	<0.25	0.50
As	<0.1	<0.1	currently not defined
Cd	<0.02	<0.03	0.50

Results are shown as the mean ± standard deviation (SD). Values in the same row that have different letters (a) differ significantly (*p* ≤ 0.05).

**Table 2:** Biogenic amines content in spiny-cheek crayfish meat.

Biogenic Amine (mg/kg)	Tryptamine	Phenylethylamine	Putrescine	Cadaverine	Histamine	Tyramin
Stari Slankamen	ND	ND	ND	ND	ND	ND
Begeč	ND	ND	ND	ND	ND	ND

Results are shown as the mean ± standard deviation (SD). ND—not detected.

**Table 3:** Macro- and micro-mineral content of spiny-cheek crayfish meat.

Minerals (mg/kg)	SV (Slankamen)	SV (Begeč)
Macro-minerals		
Ca	603.15 ± 115.90 ^a^	597.56 ± 79.57 ^a^
K	1524.50 ± 272.82 ^a^	1625 ± 337.05 ^a^
Mg	229.06 ± 53.12 ^a^	229.72 ± 22.95 ^a^
Na	1047.57 ± 38.54 ^a^	1067 ± 24.64 ^a^
P	2957.50 ± 67.18 ^a^	3560 ± 251.59 ^a^
Micro-minerals		
Fe	10.70 ± 1.39 ^a^	9.69 ± 2.85 ^a^
Cu	14.07 ± 1.70 ^a^	10.21 ± 0.83 ^a^
Zn	22.36 ± 4.56 ^a^	16.60 ± 1.83 ^a^
Mn	1.76 ± 0.71 ^a^	1.49 ± 0.76 ^a^

Results are shown as the mean ± standard deviation (SD). Values in the same row that have different letters (a) differ significantly (*p* ≤ 0.05).

**Table 4:** Chemical composition of spiny-cheek crayfish meat.

Results of Chemical Analysis	Stari Slankamen	Begeč
Energy value (kJ/100 g)	313.25 ± 16.78 ^a^	332.55 ± 40.37 ^a^
Energy value (kcal/100 g)	73.90 ± 3.76 ^a^	78.00 ± 8.49 ^a^
Moisture content (%)	80.48 ± 1.08 ^a^	79.48 ± 2.29 ^a^
Ash content (%)	1.33 ± 0.30 ^a^	1.21 ± 0.06 ^a^
Protein content (%)	16.45 ± 2.74 ^a^	16.90 ± 1.22 ^a^
Fat content (%)	0.20 ± 0.06 ^a^	0.23 ± 0.12 ^a^
Total carbohydrates (%)	1.54 ± 2.02 ^a^	2.19 ± 1.11 ^a^
Of which sugars (%)	1.15 ^a^	0.78 ± 0.34 ^a^

Results are shown as the mean ± standard deviation (SD). Values in the same row that have different letters (a) differ significantly (*p* ≤ 0.05).

**Table 5:** Amino acid profile from spiny-cheek crayfish meat.

Amino Acid (g/100 g Sample)	Stari Slankamen	Begeč
Threonine *	0.75 ± 0.04 ^a^	0.71 ± 0.01 ^a^
Valine *	0.84 ± 0.03 ^a^	0.85 ± 0.01 ^a^
Methionine *	0.59 ± 0.11 ^a^	0.47 ± 0.00 ^a^
Isoleucine *	0.76 ± 0.04 ^a^	0.77 ± 0.01 ^a^
Leucine *	1.24 ± 0.03 ^a^	1.31 ± 0.03 ^a^
Phenylalanine *	0.77 ± 0.07 ^a^	0.74 ± 0.00 ^a^
Histidine *	0.32 ± 0.02 ^a^	0.31 ± 0.00 ^a^
Lysine *	1.61 ± 0.04 ^a^	1.64 ± 0.02 ^a^
Aspartic acid	1.94 ± 0.10 ^a^	2.07 ± 0.02 ^a^
Serine	0.86 ± 0.05 ^a^	0.85 ± 0.00 ^a^
Glutamic acid	2.51 ± 0.15 ^a^	2.77 ± 0.01 ^a^
Proline	0.42 ± 0.02 ^a^	0.34 ± 0.00 ^b^
Glycine	0.83 ± 0.02 ^a^	0.88 ± 0.00 ^a^
Alanine	0.90 ± 0.00 ^a^	0.97 ± 0.00 ^b^
Cystine	0.34 ± 0.02	ND
Tyrosine	0.62 ± 0.04 ^a^	0.60 ± 0.01 ^a^
Arginine	1.37 ± 0.03 ^a^	1.46 ± 0.02 ^a^
TAA	16.50 ± 0.84 ^a^	16.73 ± 0.03 ^a^
TEAA	6.88 ± 0.30 ^a^	6.78 ± 0.06 ^a^
TNEAA	9.63 ± 0.55 ^a^	9.93 ± 0.04 ^a^

Results are shown as the mean ± standard deviation (SD). Values in the same row that have different letters (a, b) differ significantly (*p* ≤ 0.05). Essential amino acids are indicated by an asterisk (*). Abbreviations: TAA, total amino acids; TEAA, total essential amino acids: TNEAA, total nonessential amino acids; ND, not detected.

**Table 6:** Fatty acid profile from spiny-cheek crayfish meat.

Fatty Acids	Stari Slankamen (g/100 g Total Fatty Acids)	Begeč (g/100 g Total Fatty Acids)
C 10:0	0.2 ± 0 ^a^	1.3 ± 0 ^b^
C 12:0	0.1 ± 0	ND
C 14:0	0.82 ± 0.35 ^a^	9.5 ± 0.1 ^b^
C 15:0	0.66 ± 0.06 ^a^	1.25 ± 0.05 ^b^
C15:1	0.52 ± 0.50	ND
C 16:0	16.503 ± 1.46 ^a^	18.30 ± 0.26 ^a^
C 16:1	4.64 ± 0.92 ^a^	5.25 ± 0.08 ^a^
C 17:0	1.07 ± 0.29 ^a^	1.03 ± 0.03 ^a^
C 17:1	0.33 ± 0.06	ND
C 18:0	6.81 ± 0.21 ^a^	11.07 ± 0.19 ^b^
C 18:1n9c	21.14 ± 0.69 ^a^	17.96 ± 0.27 ^b^
C 18:2n6c	5.51 ± 0.73 ^a^	2.14 ± 0.01 ^b^
C 18:3n3	1.27 ± 1.34 ^a^	2.13 ± 0.01 ^a^
C 20:0	0.887 ± 0.59	ND
C 20:1n9	1.22 ± 0.09	ND
C 20:3	2.00	ND
C 20:2n6	1.97 ± 0.01 ^a^	1.36 ± 0.02 ^a^
C 20:4n6	9.42 ± 0.95 ^a^	3.9 ± 0.2 ^b^
C 20:5n3 (EPA)	24.72 ± 1.01 ^a^	20.05 ± 0.45 ^b^
C 22:6n3 (DHA)	3.62 ± 0.49 ^a^	3.9 ± 0.1 ^a^
Total saturated fatty acids (SFA)	26.84 ± 1.96 ^a^	42.45 ± 0.25 ^b^
Total monounsaturated fatty acids (MUFA)	27.28 ± 1.26 ^a^	23.21 ± 0.19 ^b^
Total polyunsaturated fatty acids (PUFA)	46.54 ± 0.26 ^a^	33.47 ± 0.78 ^b^
Total omega-3 fatty acids (n-3 PUFA)	29.63 ± 0.26 ^a^	26.08 ± 0.56 ^b^
Total omega-6 fatty acids (n-6 PUFA)	16.91 ± 0.24 ^a^	7.39 ± 0.22 ^b^
(n-6)/(n-3)	0.57 ± 0.01 ^a^	0.28 ± 0.02 ^b^
(n-3)/(n-6)	1.75 ± 0.03 ^a^	3.53 ± 0.03 ^b^

Values in the same row that have different letters (a, b) differ significantly (*p* ≤ 0.05). ND, not detected.

**Table 8:** Spearman’s rank correlation coefficients between socio-demographic characteristics.

	Q2	Q3	Q4	Q5	Q6	Q7
Q1	−0.0328	−0.060	0.0436	0.117 *	0.013	−0.024
Q2		0.116 *	0.311 **	0.032	0.117 *	0.040
Q3			−0.354 **	0.095	0.012	0.216 **
Q4				−0.100	0.059	−0.170 **
Q5					−0.179 **	0.173 **
Q6						−0.271 **

* Coefficients are significant at *p* < 0.05; ** Coefficients are significant at *p* < 0.01.

**Table 9:** Respondents’ exposure to science data.

Question Number		n					Percentage				
Q12	Is your job in any way connected with scientific research or an institution?										
	Yes	216					68.6				
	No	99					31.4				
Question number	How often are you in contact with science and science research with media listed down below?										
		n					Percentage				
		1	2	3	4	5	1	2	3	4	5
Q13	Television (1–5)	92	83	80	43	17	29.2	26.3	25.4	13.7	5.4
Q14	Radio (1–5)	196	59	40	16	4	62.2	18.7	12.7	5.1	1.3
Q15	Daily/Weekly newspaper (1–5)	180	62	48	13	12	57.1	19.7	15.2	4.1	3.8
Q16	Scientific journals (1–5)	82	33	41	31	128	26.0	10.5	13.0	9.8	40.6
Q17	Internet (1–5)	21	18	50	68	158	6.7	5.7	15.9	21.6	50.2
Q18	Website from science institution (1–5)	49	39	86	60	80	15.6	12.4	27.3	19.0	25.4
Q19	YouTube or similar platform (1–5)	61	60	86	58	49	19.4	19.0	27.3	18.4	15.6
Q20	Facebook (1–5)	124	46	72	40	32	39.4	14.6	22.9	12.7	10.2
	How often are you in contact with science and science research during?										
		n					Percentage				
		1	2	3	4	5	1.0	2.0	3.0	4.0	5.0
Q21	Visits to museums and events that promote science (researchers’ night, science festival…) (1–5)	71	85	84	41	34	22.5	27.0	26.7	13.0	10.8
Q22	Visiting a zoo, botanical garden, etc. (1–5)	92	83	80	43	17	29.2	26.3	25.4	13.7	5.4
Q23	Attending events where science is discussed (1–5)	100	61	79	48	27	31.7	19.4	25.1	15.2	8.6
Q24	Reading scientific papers and books (1–5)	73	38	47	44	113	23.2	12.1	14.9	14.0	35.9
Q25	Going to the cinema and watching science-related movies (1–5)	112	77	81	29	16	35.6	24.4	25.7	9.2	5.1
Q26	Discussing science with friends/family members (1–5)	29	67	79	76	64	9.2	21.3	25.1	24.1	20.3

**Table 10:** Spearman’s rank correlation coefficients between respondents’ engagement with science.

	Q13	Q14	Q15	Q16	Q17	Q18	Q19	Q20	Q21	Q22	Q23	Q24	Q25	Q26
Q12	−0.003	−0.033	0.0009	−0.605 **	−0.483 **	−0.461 **	−0.242 **	−0.156 **	−0.279 **	−0.157 **	−0.473 **	−0.633 **	−0.1009	−0.462 **
Q13		0.352 **	0.352 **	0.085	0.155 **	0.150 **	0.285 **	0.236 **	0.195 **	0.250 **	0.117 *	0.0480	0.194 **	0.162 **
Q14			0.430 **	0.104	0.079	0.203 **	0.298 **	0.316 **	0.209 **	0.217 **	0.190 **	0.060	0.247 **	0.117 *
Q15				0.162 **	0.098	0.219 **	0.204 **	0.210 **	0.127 *	0.209 **	0.149 **	0.078	0.162 **	0.052
Q16					0.644 **	0.560 **	0.277 **	0.174 **	0.342 **	0.208 **	0.514 **	0.823 **	0.211 **	0.571 **
Q17						0.536 **	0.454 **	0.301 **	0.310 **	0.266 **	0.385 **	0.594 **	0.245 **	0.550 **
Q18							0.383 **	0.263 **	0.314 **	0.221 **	0.455 **	0.512 **	0.231 **	0.472 **
Q19								0.407 **	0.326 **	0.259 **	0.306 **	0.275 **	0.311 **	0.399 **
Q20									0.273 **	0.271 **	0.254 **	0.136 *	0.250 **	0.239 **
Q21										0.472 **	0.435 **	0.358 **	0.337 **	0.423 **
Q22											0.248 **	0.148 **	0.286 **	0.272 **
Q23												0.560 **	0.289 **	0.496 **
Q24													0.214 **	0.594 **
Q25														0.295 **

* Coefficients are significant at *p* < 0.05; ** Coefficients are significant at *p* < 0.01.

**Table 11:** Perception and awareness of biodiversity and invasive species in Serbia data.

Question Number		n	Percentage
Q31	Do you think there is enough discussion and effort to raise awareness about biodiversity conservation?		
	Yes, certainly.	8	2.5
	Yes, but not enough.	162	51.4
	No	125	39.7
	I don’t know	20	6.3
Q32	The term “invasive species” refers to:		
	All plant species that have been deliberately or accidentally introduced from their native environment to a new habitat and have rapidly proliferated due to favorable conditions.	11	3.5
	All animal species that have been deliberately or accidentally introduced from their native environment to a new habitat and have rapidly proliferated due to favorable conditions.	19	6
	Plant and animal species that have been intentionally or unintentionally introduced from their natural habitat to a new habitat and, due to favorable conditions, have started to spread very rapidly.	250	79.4
	I don’t know.	35	11.1
Q33	Are you aware of the invasive spiny-cheek crayfish (*Faxonius limosus*) which has been found in the Danube?		
	Yes	64	20.3
	No	251	79.7
	This crayfish species can affect (mark all correct answers):		
Q34_1	The extinction of native crayfish species, such as *Astacus astacus* and *Astacus leptodactylus*	101	21.9
Q34_2	Improvement of the Danube’s biodiversity	28	6.1
Q34_3	The destruction of fish eggs and juveniles	86	18.7
Q34_4	Transmission of crayfish plague	39	8.5
Q34_5	Destabilization of riverbanks	26	5.6
Q34_6	I don’t know	181	39.3
Q35	Is Serbia’s population actively engaged in biodiversity conservation efforts?		
	Yes, of course.	2	0.6
	Yes, but not enough.	112	35.6
	No	146	46.3
	I don’t know	55	17.5
Q36	In your opinion, which gender plays a more active role in biodiversity conservation efforts in Serbia?		
	Men	26	8.3
	Women	74	23.5
	I don’t know	215	68.3
Q37	Do you believe that the biodiversity of the Danube is under threat?		
	Yes	175	55.6
	Partially, yes	82	26
	No	1	0.3
	I don’t know	57	18.1

**Table 12:** Spearman’s rank correlation coefficients between perception and awareness of biodiversity and invasive species in Serbia.

	Q32	Q33	Q34_1	Q34_2	Q34_3	Q34_4	Q34_5	Q34_6	Q35	Q36	Q37
Q31	0.186 **	0.124 *	−0.120 *	−0.048	−0.132 *	−0.095	−0.029	0.168 **	0.299 **	0.148 **	0.015
Q32		0.090	−0.057	−0.11 *	−0.054	−0.014	−0.036	0.131 *	0.116 *	0.100	0.015
Q33			−0.481 **	0.019	−0.381 **	−0.481 **	−0.192 **	0.459 **	0.082	−0.047	0.087
Q34_1				−0.046	0.653 **	0.535 **	0.401 **	−0.720 **	−0.110	0.077	−0.092
Q34_2					−0.016	0.050	0.066	−0.332 **	−0.09	−0.058	0.060
Q34_3						0.536 **	0.450 **	−0.645 **	−0.108	−0.004	−0.125 *
Q34_4							0.500 **	−0.409 **	−0.085	−0.011	−0.053
Q34_5								−0.316 **	0.009	−0.016	−0.100
Q34_6									0.161 **	−0.028	0.075
Q35										0.151 **	0.209 **
Q36											0.092

* Coefficients are significant at *p* < 0.05; ** Coefficients are significant at *p* < 0.01.

**Table 13:** Consumer attitude towards river and crayfish products.

Question Number		n	Percentage
Q40	Do you enjoy consuming river-sourced products (fish, crustaceans, etc.)?		
	Yes	138	43.8
	Partially	112	35.6
	No	65	20.6
Q41	How often do you consume river products?		
	I don’t consume them at all.	73	23.2
	Once a week	225	71.4
	2–3 times per week	14	4.4
	More than 3 times per week	3	1
	Which types of river products do you consume?		
Q42_1	Trout	201	36.6
Q42_1	Catfish	111	20.2
Q42_2	Carp	174	31.7
Q42_4	River crayfish	9	1.6
Q42_5	None	54	9.8
Q43	Would you participate in a sensory evaluation of newly developed products based on river crayfish meat for scientific research purposes? (Likert’s scale 1–5)		
	Yes, I would be happy to participate.	109	34.6
	Probably yes	46	14.6
	Not sure/Neutral	44	14
	Probably no	39	12.4
	No, I wouldn’t.	77	24.4
Q44	Would you participate in a study involving the consumption of various river crayfish dishes for their promotion during a gastronomic day? (Likert’s scale 1–5)		
	Yes, I would be happy to participate.	104	33
	Probably yes	44	14
	Not sure/Neutral	44	14
	Probably no	39	12.4
	No, I wouldn’t.	84	26.7
Q45	Would you purchase a new product based on river crayfish meat if it were available on the market? (Likert’s scale 1–5)		
	Yes, very gladly.	76	24
	Probably yes	42	13.3
	Not sure/Neutral	81	25.7
	Probably no	36	11.4
	No, I wouldn’t.	80	25.4

**Table 14:** Spearman’s rank correlation coefficients between Consumer attitude towards river and crayfish products.

	Q41	Q42_1	Q42_2	Q42_3	Q42_4	Q42_5	Q43	Q44	Q45
Q40	−0.582 **	−0.371 **	−0.326 **	−0.430 **	−0.180 **	0.548 **	−0.406 **	−0.507 **	−0.549 **
Q41		0.465 **	0.311 **	0.444 **	0.196 **	−0.714 **	0.374 **	0.415 **	0.397 **
Q42_1			0.234 **	0.170 **	0.047	−0.576 **	0.292 **	0.321 **	0.302 **
Q42_2				0.365 **	0.144 *	−0.312 **	0.249 **	0.243 **	0.194 **
Q42_3					0.109	−0.480 **	0.268 **	0.246 **	0.25 **
Q42_4						−0.026	0.201 **	0.189 **	0.219 **
Q42_5							−0.330 **	−0.366 **	−0.364 **
Q43								0.832 **	0.660 **
Q44									0.742 **

* Coefficients are significant at *p* < 0.05; ** Coefficients are significant at *p* < 0.01.

## Data Availability

Survey participants consent was waived because the survey was conducted anonymously and did not involve the collection of any personal data from respondents. Therefore, this type of research does not require specific approval from an Ethics Committee in Serbia, where the study was conducted, as it complies with the national Law on Personal Data Protection (Official Gazette of the Republic of Serbia, No. 97/08). This law is harmonized with the standards of relevant European regulations, in particular with the EU General Data Protection Regulation (GDPR). The Law applies to the processing of personal data in the context of the activities of a controller or processor within the Republic of Serbia, regardless of whether the processing itself takes place within Serbia.
